# Gulf Arabic Noun and Verb Retrieval: What Matters?

**DOI:** 10.1007/s10936-025-10148-2

**Published:** 2025-05-26

**Authors:** Tariq Khwaileh, Eiman Mustafawi, Samawiyah Ulde, Noora Essa AlAnsari, Yusuf Albustanji

**Affiliations:** 1 Department of English Literature and Linguistics, College of Arts and Sciences, Qatar University, P.O. Box 2713, Doha, Qatar; 2 Johns Hopkins Aramco Healthcare, P.O. Box 31311, Dhahran, Saudi Arabia

**Keywords:** Arabic, Morphology, Morphological factors, Nouns and verbs, Word retrieval, Noun retrieval, Verb retrieval, Lexical processing

## Abstract

Differential processing between the grammatical classes, i.e., nouns and verbs, has been studied across various linguistic disciplines in different languages, but not Arabic. The present study explores predictors of bare single nouns and bare single verbs in Gulf Arabic through a picture-naming paradigm. Aspects specific to the morpho-phonology of the language (CV skeleton, vocalic pattern) have been investigated for their roles in noun and verb retrieval. A picture-naming paradigm was carried out with 64 healthy native speakers of Gulf Arabic where participants named 282 line drawings representing nouns, and 154 line drawings representing verbs to generate naming latencies for the nouns and verbs in question. Linear regression models were fitted to analyse the relationship between grammatical class and naming latencies as well as morphological features and naming latencies. Verbs, which are more morphologically complex than nouns, were found to have higher naming latencies. Both CV skeleton and vocalic pattern impacted naming latencies and can account for the difference between verb and noun production. The results are discussed in relation to the non-concatenative morphology framework for Semitic languages.

## Introduction

Psycholinguistic investigations of nouns and verbs have reported differential processing of the two major lexical classes in many languages while investigating various factors that govern noun and verb retrieval (e.g., Levelt, 1989; Pickering and Branigan, 1998; Damasio and Tranel 1993, Daniele et al., 1994; Sereno, 1999; Day, 1979; Deutsch et al. 1998; Faroqi-Shah and Waked 2010; Kauschke and Stenneken 2008; Rosler et al. 2001). Whilst these studies are vital to the understanding of word processing, they do not take into account morpho-phonological variances among less-studied languages such as Arabic. Models of Arabic morphology fall into two overarching frameworks, the non-concatenative view (McCarthy, 1981), and the concatenative view (Benmamoun, 1999, 2003; Heath, 2003).

Both frameworks make diverging predictions about whether or not naming latencies for nouns and verbs should differ. Specifically, since the non-concatenative view posits that morphological units such as the root, CV skeleton and vocalic pattern encode semantic and grammatical information, therefore naming latencies are predicted to increase with increasing morphological complexity. Additionally, morphological complexity is expected to vary by grammatical class. While nouns only inflect for gender and number, verbs are considered more complex forms as they also inflect for information of tense and aspect. However, as the concatenative view supposes that these units do not carry any grammatical information, naming latencies are not expected to show sensitivity to differing complexity or grammatical class under this view.

## Background

### Non-Concatenative View

According to this view, Arabic is a non-concatenative language. The difference between non-concatenative and concatenative morphology is considerable. The former builds words using the morphological processes of affixation and alteration to a consonantal root, a CV skeleton and vocalic pattern (Boudelaa and Marslen-Wilson, 2001), while the latter adopts the strategy of linear affixation in word formation, which is the case in English (Boudelaa 2015; Frost et al., 1997; Mimouni et al. 1998). A consonantal root is known as a unitary entity that has no further internal structure, and it conveys semantic meaning (Boudelaa and Marslen-Wilson, 2004). Whereas CV skeleton is a sequence of consonant and vowel; it is “an abstract prosodic unit which has no surface phonetic content, it codes the phonological shape of the surface word and its primary syntactic function” (McCarthy, 1981, p. 376). Vocalic pattern carries morpho-syntactic information, and describes the sequence of vowels specified by the vocalic pattern. (Boudelaa and Marslen-Wilson, 2004). For example, the word [kitaab] ‘book’ has the CV skeleton [CVCVVC], consonantal root /ktb/ and the vocalic pattern /-i-aa-/. The consonantal root, CV skeleton and vocalic pattern respectively account for (1) conveying the semantic field, (2) determining the morphological and phonological structure of the word, and (3) providing grammatical information such as tense, number, and gender.

### Concatenative View

Benmammoun (1999, 2003) and Heath (2003) proposed an alternative view on Arabic morphology. They argue against the grammatical status of the vocalic pattern and state that vocalic patterns cannot be carrying any grammar of tense and aspect in verbs or numbers in nominal patterns. In this view, the case of number in Arabic nouns is used as evidence, by proposing that singular nouns in Arabic are not marked by any morphemes and it cannot be argued that there is a default singular pattern since singular nouns’ vocalic patterns vary in an unpredictable way, arguing against the assumption that vocalic patterns bear grammatical semantics. The second counter-argument is the problem of transfer in which roots only share morpho-phonological features with lexically related items. They provide a number of examples that are exceptional to this assumption. This view supports a word-stem view; in their view, Arabic words are no different than words from other non-Semitic languages, they are composed of lexemes and morphemes and they are formed through adding and subtracting computations. However, for Arabic it may require a different computation since the derivational implementation for Arabic is different to that of English, for example. Instead of affixing a whole word, in Arabic the prosodic part of the word is affixed. Other than that, both anchor by using words and lexemes.

### Experimental Evidence

There are numerous studies that have found evidence that surface forms in Arabic are represented cognitively on a morphemic basis, meaning that vocalic patterns and roots are considered independent morphemes. These studies report that consonantal roots and vocalic patterns play an important role in lexical access and in the structure of lexical representation in typical as well as atypical language performance (Boudelaa and Marslen-Wilson 2000, 2001, 2004, 2010, 2011; Béland and Mimouni 2001; Khwaileh, 2011; Khwaileh et al., 2015, 2017; Idrissi and Kehayia 2004; Idrissi et al., 2002; Mimouni et al. 1998; Mimouni and Jarema 1997). The consonantal root and pattern approach have been recognized to be an important and dominant theory driving Arabic neurolinguistic and psycholinguistic research. According to Boudelaa and Marslen-Wilson (2013), priming effect occurs between nouns and verbs sharing the consonantal root, for example /kitaab/ ‘book’ and /katab/ ‘wrote’. Further, CV skeletons also have a priming impact, this is illustrated in pairs such as /tiӡaaratun/ ‘trade’ and /t^ʕ^ibaaʕatun*/ ‘typography’,* these facilitate each other by sharing the verbal vocalic pattern [_i_aa_atun] (Boudelaa and Marslen-Wilson, 2013). It should follow that CV skeletons, consonantal roots and vocalic patterns may impact processing times in object spoken naming and action spoken naming.

### The Present Study

Given that morphological features such as consonantal roots, CV skeletons and vocalic patterns vary by grammatical class such that verbs encode more complex information, this should reflect in longer naming latencies for verbs than nouns. The present study will test for such an effect. Further, it will test whether this difference is predicted by the effect of the morphological features mentioned above.

The aim of this study then is to test whether naming latencies increase with increase in morphological complexity by looking at the impact of vocalic pattern and CV skeleton on naming latencies. Specifically, the following two questions are asked: are naming latencies for verbs higher than nouns? Can this difference in naming latencies be attributed to non-concatenative morphological features of the words such as CV skeletons and vocalic patterns?

## Materials and Methods

### Materials

The stimuli were extracted from the Gulf Arabic normative database (Khwaileh et al., 2018), which included 319 line drawings representing concrete nouns, and 170 line drawings representing action verbs. The discrepancy in numbers was governed by what was available in the databases. All named verbs were in the imperfective form, whereas all nouns were in the singular form, (i.e., default forms). Only nouns and verbs with high name agreement above 80%, as reported in Khwaileh et al. (2018), were included in the experiment.

The first stage of selecting the stimuli was to identify the consonantal root, CV Skeleton, and vocalic patterns for each item in the database. The purpose was to find groups of nouns and/or verbs sharing similar consonantal roots, CV skeletons, or vocalic patterns, to group items according to their roots, skeletons or patterns. Within both nouns and verbs, the consonantal roots were so diverse and hence no words sharing consonantal roots were found in the dataset, which meant that we could not include consonantal roots in our analysis. The reason for not finding items sharing consonantal roots can be attributed to the fact that Khwaileh et al.’s (2018) dataset avoided including items that are semantically related to avoid priming effects during their data collection, hence the high variability of consonantal roots in that dataset.

Within nouns, 6 different CV skeletons and 3 vocalic patterns were found to reoccur. Within the verbs, 3 different CV skeletons and 2 different vocalic patterns were found. A threshold of 15 items was set to be included, meaning that for a vocalic pattern or a CV skeleton to be included in the current data analysis, it had to occur in the database a minimum of 15 times. Tables 1 and 2 show the types and numbers of items within each grammatical class that were included in the current design and analyses.

**Table 1:** CV skeletons and vocalic patterns of nouns

CV Skeleton^a^	Number of items	Vocalic pattern	Number of items
CV:C	27	_a_ _a	21
CCV:C	20	_a_ _a:_a	17
CVCVC	44	_a_a_	18
CVCCV	24		
CVCCV:C	32		
CVCCV:CV	26		
Total:	173	Total:	56

^a^Class II CVCCVC,

Class III CVVCVC,

Class IV absent from GA and instead class I and II forms of the verb are used. Class II and III are derived from class I. (p. 40-??)

class V tCVCCVC. is derived from class II, basically reflective of class II, using prefix

Class VI tCVVCVC,are derived from class III by prefixing t-, therefore,

class VII n-CVCVC. are formed from transitive class I by adding prefix

Class VIII, CtVCVC (ntibah). Or CtVVC (rtaaH)verbs are formed from class I by infixing -t- after the first root consonant

Class IX CCVCC. verbs are formed by doubing last radical from adjectives of color or physical characteristics

Class X stVCCVC are formed by adding the prefix st- to class I

Quadrilateral CVCCVC xarbaT gaSgaS

Passive txarbaT, hence, t-CVCCVC

**Table 2:** CV skeletons and vocalic patterns for verbs

CV skeleton	Number of items	Vocalic pattern	Number of items
CVCCVC	41	_ _a_ _ɪ_	21
CCVCCV	22	_a_ _ɪ_	16
		_ɪ_ _a_	19
Total:	63	Total:	56

### Participants

The participants were 64 (34% males; 66% females) healthy native speakers of Gulf Arabic. They were all above 18 years old. All participants had completed their secondary education with Arabic as the language of instruction, and were still studying for their undergraduate/postgraduate degree at the time of the experiment. Their age range was 18–31 years old, with a mean age of 24 years. Participants were asked to sign informed consent forms, and were provided with an information sheet to explain their role in the study.

### Experimental Procedure

A picture-naming task was carried out to establish naming latencies for each selected item (182 nouns and 154 verbs). The apparatus used for the picture naming tasks consisted of the Presentation® software (Version 18.0, Neurobehavioral Systems, Inc., Berkeley, CA, www.neurobs.com), which is a response recorder. It controlled the presentation of the pictures, and automatically recorded latencies in milliseconds from the time the picture was presented until the onset of the response. If the participant did not respond within 5 s, the software presented the next stimulus. The computer automatically saved the data to an excel sheet and saved sound files of the responses. The list of stimuli was presented in a randomized manner using the auto randomization function. Participants named the object pictures first, and then named the action pictures.

The data were collected in a soundproof room, designating one session for each participant. Each session lasted for 50 to 60 min, including instructions, preparation and practice. Each session included two tasks: object naming task, then action naming task. At the beginning of the task, participants were encouraged to respond carefully and consistently to the task. Participants were given instructions and were taken through practice items prior to commencing the task in question, followed by feedback. Instructions were given in Arabic, and participants were given the opportunity to take a break, between tasks.

Participants sat at a suitable distance from a laptop screen. They were initially shown the line-drawings of objects and were asked to say out loudly the first name that comes to mind, as quickly and as accurately as possible. The researcher explained that the task was to name the object in the picture using one word only, and to avoid describing it. The same instructions were applied for the second list, which consisted of action drawings, in which the focus was to name the action being carried out in the picture, rather than the object itself, using one word only. The Presentation software used for these tasks, presented a signal in a form of a cross (+), which appeared in the centre of the screen for 1000ms. immediately followed by the picture. The cross served as a prompt to look at the centre of the screen in preparation for the upcoming picture, which remained for 5 s before the next stimulus appeared. When the participant could not recognize the picture or did not know the name of the picture, they were asked to say out loud that they could not recognize the object/action, and the researcher would take a note of the item to revisit after the experiment and delete it’s naming latency from the list. All sound files were exported to PRAAT (Boersma, Paul and Weenink, David 1992–2022: Praat: doing phonetics by computer [Computer program].Version 6.0.08. https://www.fon.hum.uva.nl/praat/), and each sound file was revisited to make sure that the software did not include false triggering of noise or ‘em’ or ‘err’. Responses were transcribed and coded by the researcher using a numerical coding system which is the coding protocol developed by Khwaileh et al. (2020), for the Arabic language.

## Results

Only pictures that were named accurately within the allotted period were scored as correct. Items with false triggers, hesitations and inaccurate responses were deleted from the data. A total of 282 noun naming latencies (mean: 1597.2 ms; std: 497.2 ms) and 154 verb naming latencies (mean: 1884.7 ms; std: 444.9 ms) were included in the analysis after cleaning the data.

Regression models were fitted and analysed using R (version 4.2.3), using the *lm* function from the lme4 package. First, a simple linear regression model was first fitted to assess the impact of grammatical class on naming latencies and test whether naming latencies are higher for verbs. Next, to test whether any observed difference can be attributed to either CV skeleton or vocalic pattern, additional simple linear regression models were fitted to assess the impact of each individual CV skeleton and vocalic pattern on naming latencies.

CV skeleton and vocalic pattern are both highly related concepts, such that certain types of vocalic patterns regularly map on to certain types of CV skeletons. However, which of the two is expected to have an effect on naming latencies is unclear. As such, both were dealt with separately to ensure no issues of multicollinearity arise. Skeletons and patterns were assigned numeric codes and were operationalized as nominal categorical variables. Simple contrast coding was applied.

The first model showed a significant effect of grammatical class on naming latencies, F (1, 467) = 14.8, RSE = 483.2, adjusted R^2^ = 0.02865, *p* = 0.000136. Specifically, verbs display significantly longer naming latencies. The coefficients of the model are reported in Table 3 below.

**Table 3:** Grammatical class ~ naming latencies

	Estimate	S.E	t	*p*
Intercept	1647.76	25.61	64.347	< 2e−16***
Verbs	201.60	52.40	3.847	0.000136***

The second model showed a significant effect of Skeleton Code on naming latencies, F (7, 231) = 2.82, RSE = 484.6, adjusted R^2^ = 0.05082, *p* = 0.007737. Further, there is significant effect of Skeleton Code 7 and Skeleton Code 8, both of which correspond to the two CV skeletons for verbs. The coefficients of the model are reported in Table 4.

**Table 4:** Skeleton codes ~ naming latencies

	Estimate	S.E	t	*p*
Intercept	1666.27	32.44	51.367	< 2e−16***
Skeleton_Code2	− 10.09	142.97	− 0.071	0.94382
Skeleton_Code3	22.65	118.47	0.191	0.84855
Skeleton_Code4	31.15	134.51	0.232	0.81703
Skeleton_Code5	123.05	126.64	0.972	0.33222
Skeleton_Code6	52.84	133.16	0.397	0.69184
Skeleton_Code7	445.95	137.51	3.243	0.00136**
Skeleton_Code8	252.51	119.54	2.112	0.03573*

The third model showed a significant effect of Vocalic Pattern on naming latencies, F (5, 106) = 2.802, RSE = 539.7, adjusted R^2^ = 0.07509, *p* = 0.02037. Further, there is significant effect of Vocalic Pattern 4, 5 and 6, all of which correspond to the three vocalic patterns for nouns. The coefficients of the model are reported in Table 5.

**Table 5:** Vocalic Patterns ~ Naming Latencies

	Estimate	S.E	t	p
Intercept	1720.44	51.26	33.561	< 2e−16 ***
Pattern_Code2	− 245.28	179.11	− 1.369	0.17375
Pattern _Code3	− 269.99	170.89	− 1.580	0.11711
Pattern _Code4	− 542.71	166.57	− 3.258	0.00151 **
Pattern_Code5	− 447.75	176.09	− 2.543	0.01244 *
Pattern _Code6	− 483.85	173.37	− 2.791	0.00623 **

## Discussion

This study tested for the effect of two morphological variables in Arabic on noun and verb naming latencies. First, we found that naming latencies for verbs are significantly longer than nouns. This is in harmony with existing studies cross-linguistically. Various studies on noun and verb processing in concatenative languages (English: (Breedin et al., 1998; Caramazza and Hillis, 1991; Shapiro and Caramazza 2003; Spanish: Yudes et al., 2016; German: Kauschke and Stenneken 2008) have found a general difference in the time required to process nouns vs. verbs. Further, nouns generally (even in concatenative languages) tend to be processed faster than their verb counterparts, this is illustrated in Mätzig et al. (2009), who examined the differences between noun and verb picture-naming and found that in both healthy and unhealthy speakers of English, object-naming elicited shorter naming latency than action-naming, they concluded that verb processing is more demanding than noun processing. This means that despite having a non-concatenative morphology as opposed to English’s concatenative one, the processing difference between nouns and verbs still holds.

This leads us to examine the nature of this difference in naming latencies. Can it perhaps be attributed to the differing morphological features of nouns and verbs? The results of this study indicate yes. The difference in naming latencies became significant once the type of vocalic pattern and CV skeleton shifted by grammatical category. This means that there is indeed a significant difference in the vocalic patterns of nouns and verbs and this difference impacts latencies; and likewise, for CV skeletons. This difference can be construed in terms of increased complexity, assuming that longer latencies correspond to increased complexity. Increased complexity of verb forms can be attributed to the fact that they inflect for additional features compared to nouns, specifically person and tense.

However, to probe further why this difference occurs, we need to examine the nature of these morphological variables. For this reason, we included each independent CV skeleton and vocalic pattern in the model to assess their individual impact. Interestingly, these variables differed significantly from each other even within grammatical class category. That is, for CV skeletons, the 2 verb skeletons (CVCCVC and CCVCCV) significantly differed from each other in how they impacted naming latencies such that naming the CCVCCV type was faster. For vocalic patterns, the 3 noun patterns (_a_ _a, _a_ _a:_a and _a_a_) were named progressively faster and the difference between them was significant. These results clearly indicate the salience of these morphological variables in impacting naming latencies.

Moving into a closer look at the difference between nouns and verbs, it is curious that CV skeletons predicted verbs and vocalic patterns predicted nouns. This pattern of determinants is at odds with what has been reported in Deutsch et al. (1998), who report vocalic patterns as facilitators of verbs but not nouns, in Hebrew. They found facilitation effects in lexical decisions and in naming when the targets and the primes shared either a vocalic pattern, or consonantal root in Hebrew verb morphology; leading them to conclude that verbal consonantal roots and vocalic patterns have a role in the lexical organization within the verbal system. However, Deutsch et al. (1998) used a different experimental paradigm to what has been used in the current dataset; they used a combination of lexical decision and naming tasks. Furthermore, they designed their stimuli from scratch to fit the purpose of their study, and hence managed to control for variables that were absent in the current study, i.e. consonantal roots.

Another study by the same group of researchers reported findings from Hebrew that are at odds with the current findings from Arabic. Frost et al.’s (1997) study looked into the priming effects of Hebrew consonantal root and vocalic patterns. They reported that in nouns, the vocalic pattern does not govern lexical retrieval, which they attributed to the large number of nominal patterns and low semantic transparency.

Boudelaa et al. (2011) explains that highly productive words have strong vocalic pattern priming effects, however; as mentioned, nouns are not as highly productive as verbs. Thus, the vocalic pattern does not significantly impact the naming latency of nouns. The effect of vocalic patterns on noun naming latencies found in the current data is not consistent with Boudelaa et al. (2004) findings. Boudelaa et al. (2004) found that vocalic patterns did not have any priming effect on lexical processing, which is harmony with the data from Hebrew described above. Both of these studies made use of priming in lexical decision tasks. Furthermore, Boudelaa and Marslen-Wilson (2011) established that Arabic vocalic patterns have a strong priming effect on highly productive word roots; and as previously mentioned, Arabic verbs are highly productive compared to nouns but in this data, vocalic patterns significantly predict nouns and not verbs. Vocalic patterns also explain the highest degree of variance in this study’s data (accounting for 7.50%) indicating the salience of this effect.

It is possible that these apparent contradictions are solely to do with modality, as all of the above-mentioned studies have investigated the effect of vocalic patterns in comprehension tasks. Given that they are fundamentally different processes, perhaps vocalic patterns have no impact on noun comprehension but do so in production. Nonetheless, the expectation was that vocalic patterns would predict verbs too, as vocalic patterns encode grammatical information we therefore expected more information to be encoded in verbal vocalic patterns. There are several possibilities for why this result might have been found. The first is that the answer for why naming latencies for verbs are longer may not necessarily lie in their increased morphological complexity. Or more specifically, that patterns of complexity are not as straightforward as to consider nouns and verbs as homogenous categories in this regard. Issa (2022) investigated morphological complexity in reading in children aged 7–14 and found varying patterns of complexity such that different types of nouns and verbs also differed in complexity. For instance, active voice verbs and augmented verbs had comparable complexity to various types of nouns (sound masculine plural noun, the sound feminine plural noun, broken plural of paucity, broken plural of multitude, relative adjective, and diminutive) whereas passive voice verbs were the most difficult.

This leads us to the second consideration, that it is possible that the effect observed is simply an artefact of the stimuli selected for this study. Since all stimuli were selected from an already existing database, the selection was governed by what was available. As mentioned in Methods above, a minimum threshold of 15 items was set, meaning that in order to be included in the study, a vocalic pattern or CV skeleton had to occur in the database a minimum of 15 times. Future studies should design the stimuli from scratch to include a wider variety of patterns and skeletons, as well as manipulate the differences between them to be representative of all the various types of nouns and verbs.

Finally, it is worth reiterating that the results clearly indicate the salience of vocalic patterns and CV skeletons as morphemic units. This study thus adds to the body of research and is in harmony with what has been reported in previous research on Semitic languages, i.e. Arabic and Hebrew (Boudelaa and Marslen-Wilson, 2000, 2001, 2004, 2010, 2011; Béland and Mimouni, 2001; Deutsch et al., 1998; Khwaileh, 2011; Khwaileh et al., 2015, 2017; Idrissi and Kehayia, 2004; Idrissi et al., 2002; Mimouni et al., 1998; Mimouni and Jarema, 1997). These studies have reported an effect of consonant root, CV skeleton and vocalic patterns on lexical processing in Semitic languages, using both healthy and neuropsychological data. As such, these results overarchingly support a non-concatenative view of Arabic morphology. In opposition to Benmammoun (1999, 2003) and Heath (2003), the salience of vocalic pattern in impacting latencies suggest it indeed carries information such that variation in this information results in different naming latencies. A non-concatenative theory of morphology typically presents a number of challenges for generative accounts of syntax, particularly for theories like Distributed Morphology, which assumes concatenative and local syntactic operations.

While a Current Consensus as described by Kastner and Tucker (2019) has been reached which resolves several aspects of non-concatenative morphology under Distributed Morphology, challenges still remain. It remains particularly unclear precisely how much morphological information needs to be present at the phonological form. The salience of vocalic patterns and CV skeletons as morphemic units as understood in this study sheds some light on this question, although further investigation is required. Further, there is little formal analysis that investigates the consequences of cross-categorical derivations beyond denominal verbs. Empirical work investigating the difference between nouns and verbs such as in this study can be particularly informative in this regard. Although a full analysis is outside the scope of this discussion, it is clear that the results of this study have implications for generative theories of morphosyntax.

While these results are not directly generalizable to populations such as those with language disorders, the results nonetheless can (a) be used to inform further investigation in disordered populations and (b) have indirect implications for clinical research and practice. Disrupted naming ability is frequently found in acquired language disorders such as aphasia and dementia. Further, dissociations between noun and verb naming also tend to occur, although the underlying mechanisms that are responsible for these disruptions remain contested. Lexical models of production (Dell et al., 1997; Garrett, 1990; Levelt et al., 1999) have been typically used to account for this dissociation. As grammatical category information is assumed to be specified at the lemma level in these models, disruptions at this stage or further along have been interpreted to cause selective deficits in either category. Some accounts have also proposed morphological complexity as a cause, suggesting that more morphologically complex forms are more prone to errors and disruption, particularly in agrammatic persons with aphasia. However, difficulty with different categories ought to reflect in latencies as well. Further studies can thus test whether longer latencies correspond to more errors in naming. This would inform not only assessment and diagnosis but therapy and interventions as well, since recovery of more morphologically complex forms will undoubtedly require special attention.

## Conclusion

The current study has assessed both noun and verb production on a single-word level and has found that Arabic verbs have significantly longer naming latencies than nouns. Further, CV Skeleton and vocalic patterns have significant effects on naming latencies and can account for the difference in verbs and nouns. Specifically, when CV skeletons and vocalic patterns switched from nouns to verbs, the latencies became significantly longer. Finally, significant differences in latencies were found even within different types of noun vocalic patterns and different types of verb CV skeletons. These results add to the body of literature that that supports a non-concatenative view of Arabic morphology under which CV skeletons and vocalic patterns can be considered as independent, information-carrying morphemic units. However, we cannot claim that the current dataset offers enough data to make a conclusive assumption on how these units interplay at granular level. This is due to the limitation in the current dataset, and the absence of crucial factors for the items investigated i.e. consonantal roots. Future studies need to include consonantal roots in their stimuli design, and include more variability of vocalic patterns and CV Skeletons than what is reported in this study which was dependent on a limited number of vocalic patterns and CV skeletons governed by what is available in the database used for this investigation.

## Data Availability

The dataset generated by and analysed in this study is available for reviewers to access under Supplementary Information.

## Appendix

### Samples

1. Nouns:

• Data Examples:

**Table:**

	English	MSA/Aralex Form	Root	CV-skeleton	Pattern	Transcription
(a)	Crescent	هلال	tˤ.b.l	ccv:c	_ _a:_	hla:l
(b)	Arrow	سهم	s.h.m	cvcvc	_a_ɪ_	sahɪm

Verbs:

• Data Examples:

**Table:**

	English	MSA/Aralex Form	Root	CV-skeleton	Pattern	Transcription
(a)	Cry	يصيح	sˤ.ħ	ccv:c	_ _i:_	jsˤi:ħ
(b)	Arrange	يرتب	r.t.b	ccvccvc	_ _a_ _ɪ_	jrattɪb
